# Theoretical Investigation on Failure Behavior of Ogive-Nose Projectile Subjected to Impact Loading

**DOI:** 10.3390/ma13235372

**Published:** 2020-11-26

**Authors:** Zhao Li, Xiangzhao Xu

**Affiliations:** State Key Laboratory of Explosion Science and Technology, Beijing Institute of Technology, Beijing 100081, China; 3120160075@bit.edu.cn

**Keywords:** impact test, structural failure, concrete slabs, theoretical model

## Abstract

Experimental and theoretical investigations on the failure behaviors of projectile during high-speed impact into concrete slabs were performed in this study. The ogive-nose projectiles after impact experiments were recovered and their microstructures were observed by scanning electron microscope and metallographic microscope. Mass abrasion and nose blunting are the typical failure models of steel projectile. Furthermore, thermal melting and cutting are the two main failure mechanisms. Based on the microscopic experimental results, a theoretical model of ogive-nose projectile subjected to impact loading considering the melting and cutting mechanisms was proposed. A modified cap model is introduced for describing the failure behavior of concrete targets, and then the dynamic cavity expansion theory is used to determine the resistance of projectiles during penetration. Besides, combining with the two-dimensional heat conduction equation and abrasive wear theory, the two main abrasion mechanisms of melting and cutting are included in the proposed model, which breaks through the framework of previous abrasion models with single abrasion mechanism. The predicted results of the present abrasion model are in good agreement with the experimental data, which indicates that the proposed model can effectively predict the failure behavior and penetration performance parameters of high-speed projectiles during penetration into concrete targets, such as mass loss, nose blunting, and depth of penetration.

## 1. Introduction

The dynamic responses and failure models of metal material under high pressure and high strain are extremely complex. Especially, the failure analysis of metal material subjected to impact loading is intractable [1,2,3,4,5]. This paper focuses on the failure behavior of metal projectile during high-speed impact into concrete slabs. Previous works have indicated that projectiles keep generally nondeformable under low-speed impact loading [6,7,8]. Thus, the metal projectiles were assumed as rigid for the theoretical analysis convenience, and the predictions agreed well with the experimental data [9,10,11]. However, extensive impact experiments were conducted and indicated that the projectiles occurred obvious structural failure, such as nose blunting and mass abrasion, during high-speed impact experiments and the mass abrasion mainly occurred in projectile nose [12,13,14,15,16,17,18,19]. Furthermore, the failure behavior of metal projectiles obviously reduces the penetration efficiency and may establish asymmetry in the nose that result in unstable motion of the projectiles. For the safety of structural members and the improvement of penetration performance, a more reasonable understanding of failure behavior of projectile when subjected to impact loading, which produces large inelastic deformations, is important.

Some researchers have focused on the structural failure of projectile considering the mass abrasion and nose blunting. Silling et al. [20] found a linear dependence between projectile mass loss and initial kinetic energy and developed a successful empirical formula to predict the mass loss and change in nose shape for steel projectiles for initial impact velocities ≤1000.0 m/s. Subsequently, Wen et al. [21] and Chen et al. [22] proposed abrasion models referring to Silling’s empirical formula. Those abrasion empirical formulas were established based on summary of extensive experimental data. However, empirical formulas do not reveal the mechanisms of mass abrasion and heavily rely on a large number of experimental data under specific experimental conditions, which limited their wider application. Thus, exploring mechanism of mass abrasion is an effective method to establish abrasion model. Jones et al. [23] believed surface melting of the nose of the projectile was the primary cause of mass abrasion owing to the microstructural observations of surface layers and assumed that all the heat generated by friction between projectile and concrete was used to melt the surface layers of nose material. The predictive expression proposed by Jones included the constant of proportionality, which is difficult to obtain and sometimes overestimates the mass loss. Then, Davis et al. [24], He et al. [25], and Ouyang et al. [26] improved the Jones model by adding parameters considering aggregate hardness or corrective factor. Besides, Guo et al. [27] indicated the wear of projectile surface material is the main factor of mass loss, and proposed a mass abrasion model based on the Archard’s theory. Recent studies [20,21,22,23,24,25,26,27,28] generally use the dynamic cavity expansion theory or semi-empirical formula to analyze the response of projectile during impact. Moreover, the linear failure criteria or equation of state (EOS) are used to describe the dynamics mechanical behavior of concrete in these studies, which are not suitable for high pressure and high strain during high-speed penetrating into concrete.

In this work, four sets of high-speed impact experiments were conducted to study the failure of projectiles caused by mass abrasion. Parameters that characterize structural failure, such as mass loss and nose shape change, and parameters that characterize penetration performance, such as the depth of penetration (DOP), were obtained, and the microstructure of the surface of residual projectiles were observed by scanning electron microscopy (SEM) and metallographic microscope. Several parallel and long grooves can be observed on the surface of the nose, which means the surface material of the projectile was cut by hard particles such as aggregates in concrete target. Meanwhile, continuous heat affected zone (HAZ) with different thickness can also observed on the longitudinal section of the residual projectile surface, which indicated that the surface material of the projectile was heated and even melted. Therefore, the above two mechanisms work simultaneously and interact with each other, and they are the two main factor of mass abrasion. Moreover, the mass abrasion of projectiles become obvious during high-speed penetration into concrete targets. In the existing works, aiming to simplify the solution procedure, the linear yield criteria, such as Tresca [29], Drucker–Prager [30], and Mohr–Coulomb criteria [11,31], as well as the linear pressure–volume strain relationship [11], were introduced for describing the mechanical behavior of concrete target. Generally, the shear strength–pressure relationship is nonlinear and the pressure–volumetric strain exhibits obvious nonlinear characteristics. Thus, the Ottosen four-parameter criterion [32] coupled with the cap yield surface and HJC model [33], which were more suitable for high pressure conditions, were adopted for describing the dynamic mechanical properties of concrete in this study. Finally, a theoretical model considering two main abrasion mechanisms was proposed to predict the failure characteristics of an abrasive projectile. The prediction results of this theoretical model agreed well with experimental data by this study and others, which verified the validity of the proposed model.

## 2. Microscopic Analysis of the Recovered Projectile

**Figure 1:**
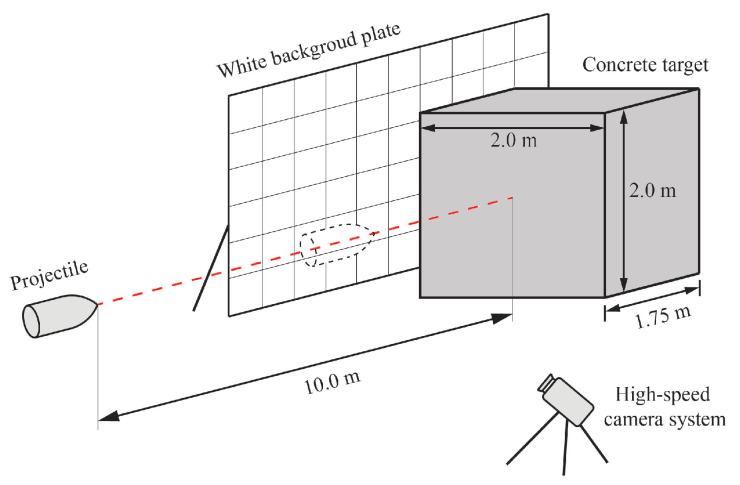
Plan sketch of experiment layout.

Four sets of high-speed impact tests were carried out to investigate abrasion mechanisms of projectiles. Ogive-nose projectiles with length $L_{p}$, diameter *d*, initial mass $m_{0}$, and caliber-radius-head (CRH) of 197.5 mm, 100.0 mm, 5.7 kg, and 1.2, respectively, were used. The high strength projectiles were machined from the PCrNi3MoV steel alloy with yield strength of 835.0 MPa. The projectiles were launched by a smooth bore powder. All of the projectiles were fired into concrete slabs prepared from the same batch and well-cured in standard conditions for 28 days. The concrete slabs have cross-section dimensions of 2.0 m by 2.0 m and a thickness of 1.75 m, as shown in Figure 1. The target dimensions are large enough to avoid lateral boundary effect, and its compressive strength $f_{c}$ and density $\rho_{t}$ were tested as 50.0 MPa and 2300.0 kg/m^3^, respectively. The high-speed cameras were set orthogonally to record and measure the flight attitude of the projectile, and all units were aligned and fixed to make sure that the projectile could hit the target normal to its surface.

**Figure 2:**
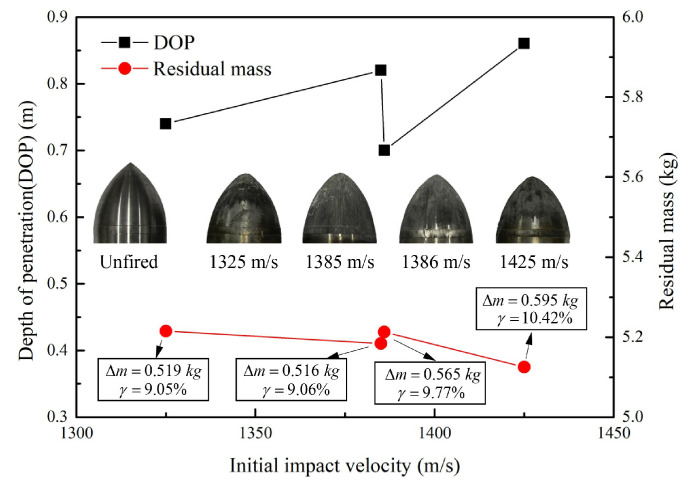
Results of impact experiments including depth of penetration *H*, mass loss Δm, mass loss rate γ, and final nose of the residual projectiles.

All the experimental conditions for both four groups of impact tests were identical except for the initial impact velocity of the projectiles. The projectiles were recorded after impact tests to study the abrasion mechanisms of projectiles during penetration. Experimental results including the DOP *H*, mass loss Δm, mass loss rate $\gamma =\Delta m/m_{0}$, and nose change of the projectiles are summarized in Figure 2.

Figure 2 shows the DOP of projectiles are generally larger with the increase of initial impact velocities, while the mass of residual projectiles becomes smaller with the increase of initial impact velocities. Particularly, the reason for the “unexpected” behavior of the specimen associated to 1386.0 m/s may be that the resistance of the projectile is not always uniform, which may result in the penetration trajectory is not kept perfectly straight, under complex experimental conditions. Besides, from the typical comparison photograph of the original projectile and the residual projectiles in Figure 2, the majority of mass abrasion occurred on the nose of projectile during high-speed penetration, as expected.

**Figure 3:**
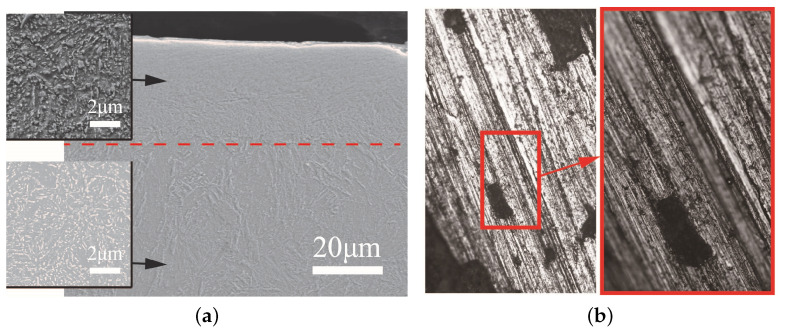
The surface microstructure of the residual projectile. (**a**) Microstructure of longitudinal section of residual projectile nose. There are two distinct zones: the microstructure inside the projectile almost unaffected by friction heat, while the microstructure near the surface of the projectile becomes denser. (**b**) Microscopic observation of the surface of the projectile nose by metallographic microscope. Many parallel and long grooves distributed on surface of the projectile nose.

After the residual projectiles were recovered, the impurities on its surface were removed firstly. Samples with the size of 10 mm × 8 mm × 10 mm were acquired from typical locations of nose residual projectiles. Then, those specimens were polished and etched by 4.0% solution of nitric acid and alcohol. Some steps such as grinding and polishing were undertaken on the observation surface of samples. Next, the prepared samples were ultrasonically cleaned in ethanol for 15 min. The S-4800 scanning electron microscope (SEM) and OLYMPUS metallographic microscope were used to observe the microstructure of samples from residual projectiles. Figure 3a shows the typical microstructure of the longitudinal sections of the residual projectiles surface. Different from the internal matrix material, a denser layer of material exists on the surface of the residual projectile, which was generally call the heat affected zone (HAZ). Besides, as shown in Figure 3b, many grooves were distributed on the surface of the residual projectile through the metallographic observation. These parallel and long grooves indicated that the hard particles, such as aggregates in concrete, cut the surface material of the projectiles during penetration. The above microscopic observation and analysis revealed that the surface material of the projectiles was softened and even melted by heat generated by friction between projectile and concrete target during penetration, and the peeling of thermal melting and cutting are the two main abrasion mechanisms.

By comparing the microstructure erosion in different locations of the recovered projectiles, mechanisms of the mass loss process are proposed to support the theoretical research in this section.

## 3. Abrasion Model

By the observation of the microstructure in nose of the recovered projectiles, the mass abrasion mechanisms are proposed to support the theoretical research in this section.

### 3.1. Constitutive Model

The concrete constitutive model consists of equation of state defining the pressure and strain relationship and the yield criterion, which describes the failure condition. According to previous work [9,10,11,13,14], the rate-independent concrete constitutive model still works well in the impact problem within a certain range of initial impact velocity. In addition, as the aim of this paper is to propose a concise engineering formula without the tedious calculation process, the rate-independent concrete constitutive model is adopted in this section. During the high speed penetration, the pressure around the projectile–target interface can reach a very large value. For convenience of analysis, the linear failure criteria and equation of state were generally adopted in dynamic cavity theory when calculating the resistance of projectile. However, the shear strength–pressure relationship and pressure–volume–strain relationship exhibit obvious nonlinear characteristics under high pressure. To better describe the dynamic mechanical behavior of concrete material during penetration, a modified cap model and a three-stage HJC equation of state [33] are adopted in existing dynamic cavity expansion theory.

As a composite material, the pressure–compaction response of concrete subjected to high pressure is very complex. The three-stage HJC equation of state is governed by [34]: The first stage is linear elastic phase, which means hydrostatic pressure and volumetric strain satisfy linear relation. $p_{e}$ is the elastic limit pressure. The second stage is referred to the transitional region where the air voids are gradually compressed out of the concrete and the plastic volumetric strain produces the compaction damage until reaching the point $p_{l}$. The third stage is defined as the region where all air voids are removed from concrete and concrete is completely dense. To simplify the calculation, hydrostatic pressure and volumetric strain are assumed to be linear in the third stage.

(1) $$\left\{\begin{array}{cc} p=K_{e}\kappa , & 0\leq p\leq p_{e}\\ p=p_{e}+K_{t}\left(\kappa -\kappa_{e}\right), & p_{e}\leq p\leq p_{l}\\ p=p_{l}+K_{l}\left(\kappa -\kappa_{l}\right), & p_{l}\leq p \end{array}\right.$$

where $K_{e}$, $K_{t}$, and $K_{l}$ are the bulk modulus in different stages, and volumetric strain $\kappa =\rho /\rho_{0}-1$. $\kappa_{e}$ and $\kappa_{l}$ are volumetric strain corresponding to $p_{e}$ and $p_{l}$, respectively.

Besides, the cap model, which originated from the Cambridge model [35], have been used primarily for geological materials such as soils, rocks, and concrete. Considering the shear and compaction of concrete, the cap model is introduced to describe the failure of concrete target by two principal segments: (1) The shear failure surface describes the shear yield and volume expansion of concrete material under low hydrostatic pressure. The Ottosen four-parameter criterion [32], which treats the relationship of shear strength-pressure as nonlinear, was adopted in this study. (2) The cap surface describes the compaction of concrete material until reaching the point $p_{b}$. Thus, a cap surface was employed in this study owing to the high hydrostatic pressure will cause concrete voids compaction and volume yield [36].

The Ottosen failure criterion is [32]:

(2) $$A^{\prime }\frac{J_{2}}{f_{c}^{2}}+\lambda \frac{\sqrt{J_{2}}}{f_{c}}+B^{\prime }\frac{I_{1}}{f_{c}}-1=0$$

$$\left\{\begin{array}{cc} \lambda =k_{1}cos\left[\frac{1}{3}arccos\left(k_{2}cos3\theta \right)\right], & cos3\theta \geq 0\\ \lambda =k_{1}cos\left[\frac{\pi }{3}-arccos\left(-k_{2}cos3\theta \right)\right], & cos3\theta <0 \end{array}\right.$$

where $A^{\prime }$, $B^{\prime }$, $k_{1}$, and $k_{2}$ are four experimental parameters measured by tests of uniaxial compressive strength, circumferential compressive strength, biaxial isobaric strength, and triaxial isobaric strength. $I_{1}$ and $J_{2}$ are the first principal invariant of stress tensor and second principal invariant of stress deviator tensor, respectively. θ=π/3 is the stress angle owing to the concrete is generally compressed during penetration. Thus, the following forms of concrete shear failure surface are obtained:

(3) $$q^{2}+a_{1}q+a_{2}p-a_{3}=0$$

(4) $$a_{1}=\frac{\sqrt{3}\lambda f_{c}}{A^{\prime }};\;a_{2}=\frac{9B^{\prime }f_{c}}{A^{\prime }};\;a_{3}=\frac{3f_{c}^{2}}{A^{\prime }}$$

where *q* and *p* are hydrostatic pressure and the von Mises equivalent stress, respectively. They can be expressed in spherical coordinate as:

(5) $$p=\frac{1}{3}I_{1}=\frac{1}{3}\left(\sigma_{r}+2\sigma_{\theta }\right)$$

(6) $$q=\sqrt{3J_{2}}=\left|\sigma_{r}-\sigma_{\theta }\right|$$

The cap yield surface provides an inelastic hardening mechanism to account for plastic compaction and helps to control volume dilatancy when the material yields in shear surface, which is written as:

(7) $$\sqrt{{\left(p-p_{m}\right)}^{2}+{\left(Rq\right)}^{2}}-R\tau_{m}=0,p_{m}<p<p_{b}$$

where $\tau_{m}$ is the peak shear strength, $p_{m}$ is an evolution parameter that represents the volumetric plastic strain caused hardening or softening, and $p_{b}$ is the hydrostatic pressure when the shear strength drops to 0. When the pressure exceeds the initial compacting stress $p_{l}$, the concrete loses its shear strength and finally becomes the material with no more densification, and $p_{b}=p_{l}$ [37,38]. *R* is a material parameter controlling the shape of the cap.

**Figure 4:**
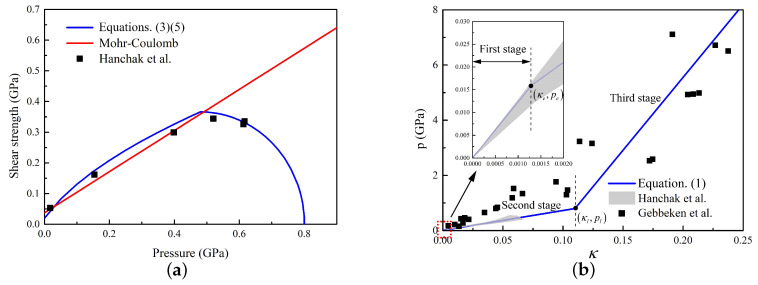
Experimental data and calibrated constitutive laws: (**a**) yield criterion [11,39]; and (**b**) equation of state [39,40].

In summary, combining Equations (1), (3), (4), and (7), the concrete constitutive law are obtained for estimating the resistance of projectile subjected to impact loading by dynamic expansion theory. Specifically, concrete with compressive strength of 48.0 MPa is chosen as an example [39]. The shear strength–pressure experimental data, cap model, and the linear Mohr–Coulomb yield criteria are presented in Figure 4a. It can be clearly observed that the cap model reproduces the tests data excellently. Besides, the experimental data from Hanckak [39] for 48.0 MPa and Gebbeken [40] for 51.2 MPa are plotted in Figure 4b. It indicates that the frequently used linear elastic EOS [10] does not agree well with the experimental data under high pressure, while the three-stage HJC equation of state has satisfactory agreement with the experimental data.

### 3.2. Equations of Projectile Motion

**Figure 5:**
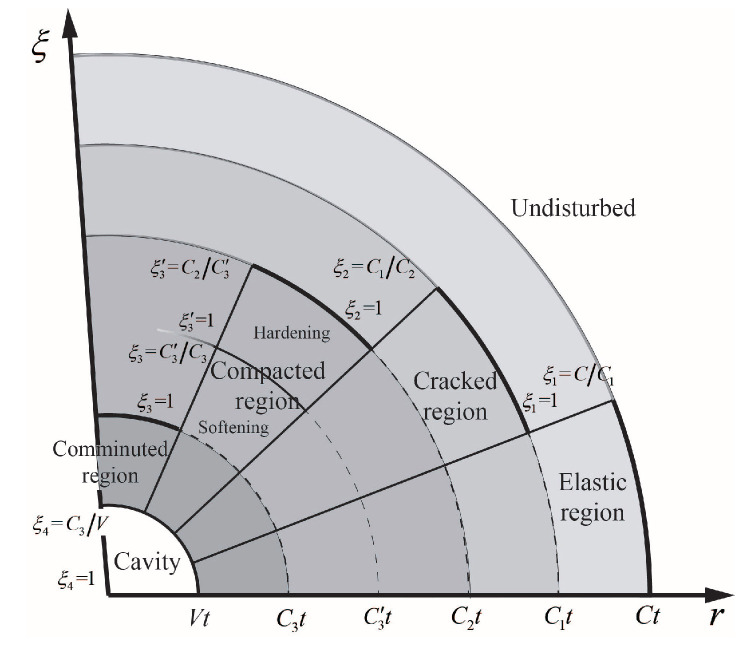
Diagram of compressible concrete media cavity expansion response regions. The interface speeds *C*, $C_{1}$, $C_{2}$, $C_{3}^{\prime }$, and $C_{3}$ and dimensionless variables *S* and ξ with different subscripts are used to designates the boundaries of different response regions.

The cavity expansion theory was used to obtain the resistance of projectile during penetration. Based on continuum mechanics, the equations of mass and momentum conservation for a compressible concrete target in Eulerian coordinates at time *t* are

(8) $$\frac{\partial \sigma_{r}}{\partial r}+\frac{2(\sigma_{r}-\sigma_{\theta })}{r}=-\rho \left(\frac{\partial v}{\partial t}+v\frac{\partial v}{\partial r}\right)$$

(9) $$\rho \left(\frac{\partial v}{\partial r}+2\frac{v}{r}\right)=-\left(\frac{\partial \rho }{\partial r}+v\frac{\partial v}{\partial r}\right)$$

where *r* is the radial coordinate, ρ is density of concrete target, *v* is partial velocity measured positive outward, and $\sigma_{r}$ and $\sigma_{\theta }$ are radial and circumferential Cauchy stress components taken positive in compression. When a spherically symmetric cavity embedded in an infinite isotropic media expands from zero initial radius at a constant velocity, spherical stress waves are generated to form different response regions corresponding to the concrete constitutive model. The response in concrete due to expansion of a cavity consists of four regions: elastic region, cracked region, compacted region, and condensed region. The material in cracked region can support only compressive radial stress. Specifically, the comminuted region corresponds to the third stage of the EOS. Meanwhile, the concrete material in compacted region is taken to behave along the shear failure surface until the cap surface is reached, which means the compacted region is divided into hardening compacted region and softening compacted region by a cut-off point $p_{m}$ based on cap model. Figure 5 shows the different response regions and designates the boundaries in terms of dimensionless variables to be introduced in subsequent sections.

Equation (6) is simplified in dimensionless form by introducing the dimensionless variables [11]

(10) $$\xi =r/c^{\prime }t;\;S=\sigma_{r}/f_{c};\;U=v/c^{\prime }$$

where $c^{\prime }$ is the interface speed between different response regions. Then, the conservation equations in dimensionless forms can be expressed as

(11) $$\frac{dU}{d\xi }=2\frac{\frac{U}{\xi }+\left(\frac{\xi -U}{1-\kappa }\right)\frac{\omega f_{2}\left(p\right)}{f_{1}^{\prime }\left(\kappa \right)\xi }}{{\left(\frac{\xi -U}{1-\kappa }\right)}^{2}\frac{\phi f_{c}\omega }{f_{1}^{\prime }\left(\kappa \right)}-1}$$

(12) $$\frac{dS}{d\xi }=2\frac{\frac{f_{2}\left(p\right)}{\xi f_{c}}+\left(\frac{\xi -U}{1-\kappa }\right)\frac{\omega^{2}U}{\xi }}{{\left(\frac{\xi -U}{1-\kappa }\right)}^{2}\frac{\phi f_{c}\omega }{f_{1}^{\prime }\left(\kappa \right)}-1}$$

where $f_{1}\left(\kappa \right)$ and $f_{2}\left(p\right)=\sigma_{r}-\sigma_{\theta }$ are the general expressions of EOS and failure criteria, $\kappa =1-\rho_{0}/\rho$ is the volume strain, and $\rho_{0}$, ρ are densities of undeformed and deformed material, respectively. Besides, $f_{1}^{\prime }=dp/d\kappa$, $\omega =c^{\prime }/c_{Y}$ and $c_{Y}=\sqrt{f_{c}/\rho_{0}}$. These two conservation equations are integrated in different response regions along with the constitutive equations and the boundary conditions to yield the field solutions for the stresses and velocities.

Satapathy [41] presented equations for the dimensionless radial stress and particle displacement in the elastic region. There are two cases:

(1) Cracked region exists and $C_{2}\leq C_{1}$; the dimensionless radial stress and particle velocity in the cracked region at the elastic–cracked interface are

(13) $$S_{2+}=1$$

(14) $$U_{2+}=\frac{f_{c}\left(1+\beta^{2}\gamma^{2}\right)}{\gamma E(\beta^{2}-1)}+\frac{2f_{c}}{E(1-\beta^{2})}+\gamma u_{1+}$$

where $\gamma =C_{1}/C_{2}$, $\beta =C_{2}/C_{cr}$, and $C_{cr}=\sqrt{E/\rho_{0}}$ is defined as bar wave speed. *E* is elastic modulus of concrete material.

(2) Cracked region disappears and $C_{2}>C_{1}$; the value of dimensionless radial stress $S_{2+}$ in the elastic region at the elastic–compacted interface is still equal to 1. The dimensionless particle velocity in the elastic region at the elastic–compacted interface is

(15) $$U_{2+}=\frac{f_{c}\left(1-\alpha \right)\left(1+2\alpha \right)\left(1+\nu \right)\left(1-2\nu \right)}{2E[\left(1+\alpha \right)\left(1-2\nu \right)+\alpha^{2}\left(1+\nu \right)]}$$

where $\alpha =C_{1}/C$ and *C* is the dilatational elastic wave speed.

The cracked and compacted response regions and the elastic and compacted response regions are all linked through the Hugoniot jump conditions; thus,

(16) $$S_{2+}=S_{2-}=1$$

We introduce the dimensionless variables in the hardening compacted region as follows:

(17) $$\xi_{3}^{\prime }=\frac{r}{C_{3}^{\prime }t};\;S=\frac{\sigma_{r}}{f_{c}};\;U=\frac{v}{C_{3}^{\prime }}$$

Based on the failure criteria in Equations (2) and (4), as well as the EOS in Equation (5),

(18) $$p={\left(\sqrt{D_{1}+\sigma_{r}}\right)}^{2}-D_{3}$$

(19) $$\omega =\frac{dp}{d\sigma_{r}}=1+\frac{D_{3}}{\sqrt{D_{2}}-\sigma_{r}}$$

(20) $$\sigma_{r}-\sigma_{\theta }=\sqrt{a_{1}+a_{2}p}-a_{3}$$

where $D_{1}=\frac{a_{1}}{a_{2}}+\frac{a_{2}}{9}+\frac{2a_{3}}{3}$, $D_{2}=\frac{a_{2}}{3\sqrt{a_{2}}}$, $D_{3}=\frac{a_{1}}{a_{2}}$. By substituting Equations (18)–(20) into Equations (11) and (12), the specific governing equation of dimensionless form in the hardening compacted region can be obtained. Besides, according to Hugoniot jump conditions, $S_{3+}^{\prime }=\left(p_{m}+2\tau_{m}/3\right)/f_{c}=S_{3-}^{\prime }$.

Then, we introduce the dimensionless variables in the softening compacted region as follows:

(21) $$\xi_{3}=\frac{r}{C_{3}t};\;S=\frac{\sigma_{r}}{f_{c}};\;U=\frac{v}{C_{3}}$$

Based on the failure criteria and the EOS in the softening compacted region,

(22) $$p={\left(\sqrt{D_{1}+\sigma_{r}}\right)}^{2}-D_{3}$$

(23) $$\begin{array}{c} \omega =\frac{dp}{d\sigma_{r}}=\frac{1}{2}{\left(\frac{{\Delta p}^{2}-p_{m}^{2}-\frac{4}{9}R^{2}\sigma_{r}^{2}}{\frac{4}{9}R^{2}+1}+\frac{2p_{m}+\frac{9}{2}R^{2}\sigma_{r}}{\frac{9}{2}R^{2}+2}\right)}^{0.5}\cdot \\ \left(\frac{4p_{m}+9R^{2}\sigma_{r}}{\frac{9}{2}R^{2}+2}\cdot \frac{R^{2}}{R^{2}+\frac{4}{9}}-\frac{2R^{2}\sigma_{r}}{R^{2}+\frac{4}{9}}\right)+\frac{R^{2}}{R^{2}+\frac{4}{9}} \end{array}$$

(24) $$\sigma_{r}-\sigma_{\theta }=\frac{\sqrt{{\Delta p}^{2}-{\left(p-p_{m}\right)}^{2}}}{R}$$

where $\Delta p=R\tau_{m}$. Similarly, the governing equation of dimensionless form in the softening compacted region can be obtained by substituting Equations (22)–(24) into Equations (11) and (12). Besides, the dimensionless Hugoniot jump conditions for compacted–comminuted interface is $S_{3+}=\left(p_{l}+2\tau_{m}/3\right)/f_{c}=S_{3-}$.

The dimensionless variables are introduced into the comminuted region as follows:

(25) $$\xi_{4}=\frac{r}{Vt};\;S=\frac{\sigma_{r}}{f_{c}};\;U=\frac{v}{V}$$

Furthermore, the failure criteria and the EOS in the comminuted region are

(26) $$p=\sigma_{r}$$

(27) $$\omega =\frac{dp}{d\sigma_{r}}=1$$

(28) $$\sigma_{r}-\sigma_{\theta }=0$$

Next, based on Equations (11), (12) and (26)–(28), the governing equation of dimensionless form in the comminuted region is obtained.

Through the above analysis, a set of governing equations with initial conditions of different response regions is established for calculating the stress and velocity on the cavity surface. However, these ordinary differential equations cannot be solved analytically, thus numerical evaluation with the Runge–Kutta method was used according to Forrestal and Tzou [11]. The details of the calculation are shown in Algorithm 1. After the whole circulation, cavity expansion velocity *V* and radial stress of cavity $\sigma_{r}$ datasets were obtained. The relation between $\sigma_{r}$ and *V* is as follows [10]:

(29) $$\frac{\sigma_{r}}{f_{c}}=A+B\left[\frac{V}{\sqrt{f_{c}/\rho_{0}}}\right]+C{\left[\frac{V}{\sqrt{f_{c}/\rho_{0}}}\right]}^{2}$$

where *A*, *B*, and *C* are dimensionless parameters, respectively. The relationship of $\sigma_{r}$ and *V* was fitted by a second-order polynomial (including a linear term) in present study referring Equation (29). Specifically, as the cavity expansion theory used to predict relationship of $\sigma_{r}$ and *V* was based on concrete constitutive model without strain rate effect and viscous response, an improvement on concrete constitutive model will be achieve for accurate prediction in a wider speed range in the future. The parameters of concrete used in calculation are shown in Table 1.
**Algorithm 1** The flow chat of cavity expansion theory.**Require:** the initial parameters of concrete material: $\rho_{0}$, $f_{c}$, $f_{t}$, $p_{m}$, $p_{l}$, $\kappa_{e}$, $\kappa_{p}$, *R*, $K_{c}$, $K_{l}$, $A^{\prime }$, $B^{\prime }$, $k_{1}$, $k_{2}$, ν;**Ensure:** Cavity surface stress and cavity expansion velocity;1:Given the value of $C_{1}$2:Based on the analytic solutions for elastic region and cracking region, the value of $C_{2}$ are obtained.3:**if**$C_{2}\leq C_{1}$**then**;4:    The dimensionless stress $S_{2+}$ and velocity $U_{2+}$ at the interface between cracked region and compacted region are obtained by Equations (13) and (14);5:**else**6:    The dimensionless stress $S_{2+}$ at the interface between cracked region and compacted region. The dimensionless velocity $U_{2+}$ at that interface is obtained by Equation (15);7:**end if**8:Calculate the values of $S_{2-}$ and $U_{2-}$ by Hugoniot jump condition. Given the value of $C_{3}^{\prime }$, calculate the values of $S_{3+}^{\prime }$ and $U_{3+}^{\prime }$ by the governing equation for the compacted region with shear saturation and Equations (18)–(20).9:**if**$U_{3+}=1$ and $p\leq p_{m}$ **then**;10:    Save the dimensionless stress $S_{3+}^{\prime }$ and velocity $U_{3+}^{\prime }$ as cavity surface stress and cavity expansion velocity;11:**else**12:    **if** $C_{3}^{\prime }\leq C_{3}$ **then**;13:        Calculate the values of $S_{3-}^{\prime }$ and $U_{3-}^{\prime }$ by Hugoniot jump condition. Given the value of $C_{3}$, calculate the values of $S_{3+}$ and $U_{3+}$ by the governing equation for the compacted region with shear saturation and Equations (22)–(24).14:    **else**15:        Make $C_{3}^{\prime }=C_{3}$ and calculate the dimensionless stress $S_{3+}$ and velocity $U_{3+}$ at the interface between compacted region with shear saturation and condensed region;16:    **end if**17:    Calculate the values of $S_{3-}$ and $U_{3-}$ by Hugoniot jump condition. Calculate the values of $S_{4+}$ and $U_{4+}$ by the governing equation for the condensed and Equations (26)–(28);18:    **if** $U_{4+}=1$ and $p\leq p_{m}$ **then**;19:        Save the dimensionless stress $S_{4+}$ and velocity $U_{4+}$ as cavity surface stress and cavity expansion velocity;20:    **end if**21:**end if**

### 3.3. Mass Abrasion

For the convenience of theoretical analysis, the ballistic trajectory of the projectile keeps straight and deflect is neglected under impact. The process of penetration is generally divided into two parts: crater stage (0<H<2d) and tunnel stage (2d<H) [10]. The axial resistance on the projectile nose during crater stage is [10]:

(30) $$F_{z}=cH$$

**Figure 6:**
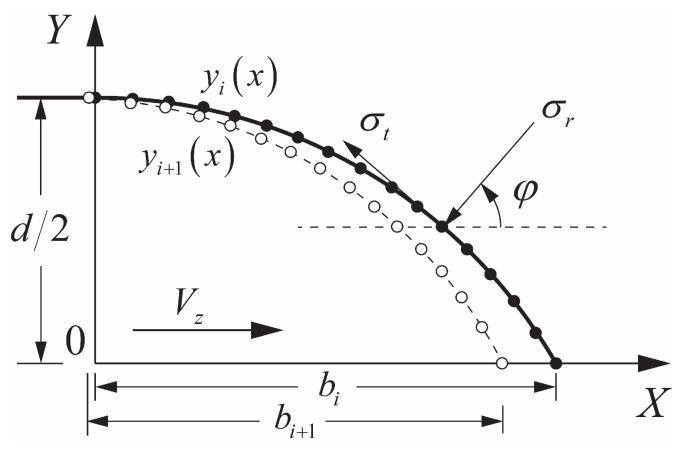
Diagram of the projectile’s nose during penetration. The solid line represents the projectile profile $y_{i}\left(x\right)$ at time step $t_{i}$ and the dotted line represent the projectile profile $y_{i+1}\left(x\right)$ at the subsequent time step $t_{i+1}$.

The mass abrasion of projectile was neglected during crater stage owing to its relatively short duration of time and displacement [42]. Define the instantaneous velocity of projectile as $V_{z}=V_{1}$ at the end of the crater stage. Considering the symmetry of the ogive-nose projectile, the section of the quarter model of projectile nose was taken to analyze the Cartesian coordinate system established as shown in Figure 6. Then, the axial resistance on the projectile nose in the tunnel stage can be expressed as [10]:

(31) $$\begin{array}{c} F_{z}=2\pi \int_{0}^{b}y\frac{cos\varphi +\mu sin\varphi }{sin\varphi }\sigma_{r}\left(V\right)\;dx\\ =2\pi (G_{0}Af_{c}+G_{1}B\sqrt{\rho_{0}f_{c}}V_{z}+G_{2}C\rho_{0}V_{z}^{2}) \end{array}$$

where $V=V_{z}cos\varphi$, φ is the angle between the normal direction of the projectile surface and the penetration direction, *b* is the length of nose, and y=y(x) is the functional expression of the outline of nose. The geometric diagram of projectile nose during penetration is shown in Figure 6. $G_{0}$, $G_{1}$, and $G_{2}$ are dimensionless shape parameters related to the friction coefficient μ and the shape of projectile nose, which are expressed as follows:

(32) $$G_{0}=\int_{0}^{b}\left(\mu y-yy^{\prime }\right)\;dx$$

(33) $$G_{1}=\int_{0}^{b}\frac{y^{\prime }}{\sqrt{1+{y^{\prime }}^{2}}}\left(yy^{\prime }-\mu y\right)\;dx$$

(34) $$G_{2}=\int_{0}^{b}\frac{\left(\mu y-yy^{\prime }\right){y^{\prime }}^{2}}{1+{y^{\prime }}^{2}}\;dx$$

According to the metallographic observation of residual projectile after penetration, it is obviously thought that the mass loss mainly comes from the peeling of molten surface layer and on projectile nose and the peeling of projectile material cutting by aggregates. In this study, the analysis of heat conduction was based on some assumptions for convenience of calculation: (1). All the heat generated by friction between projectile and target were used to melt the projectile material. (2) The thermodynamic parameters of the projectile were constant during penetration. Based on these assumptions, the two-dimensional heat conduction in projectile side of the penetration system can be expressed by the following equations.

(35) $$\rho_{p}c_{p}\frac{\partial T}{\partial t}=\lambda \left(\frac{\partial^{2}T}{\partial x^{2}}+\frac{\partial^{2}T}{\partial y^{2}}\right)$$

where *T* is the surface temperature of projectile. $\rho_{p}$, $c_{p}$, and λ are density, specific heat capacity, and heat conductivity of projectile, respectively. The initial value condition and boundary condition for solving above partial differential equation are: $T_{0}=298.0K$ and $Q\left(x,y,0\right)=\lambda \frac{\partial T}{\partial \bar{n}}$, where $Q=\mu \sigma_{n}v_{t}$ is the heat flux density of boundary, $v_{t}=V_{z}sin\varphi$ is the relative velocity between projectile and concrete target, and $\bar{n}$ is the normal direction of the nose surface. Then, the temperature distribution of projectile surface can be obtained by the two-dimensional heat conduction equation. Then, the area where the temperature exceeds the melting point $T_{m}$ of the projectile material is denoted as $\Delta S_{m}$ in any increment of time Δt.

Actually, the cutting mechanism and thermal melt mechanism worked simultaneously during penetration. More in detail, the morphology of grooves distributed on the surface of residual projectile nose were similar to abrasive wear according to microscopic observation. Therefore, a classic abrasive wear theory [43] was introduced to simulate the mechanism of cutting by aggregates. The cutting depth function g(x) can be expressed as

(36) $$g\left(x\right)=K\frac{\sigma_{r}v_{t}}{Y}$$

where *K* is the wear parameter, $v_{t}=V_{z}sin\varphi$ is the relative velocity between projectile and concrete target, and *Y* is the dynamic yield strength of surface material of projectile. Then, the area cutting by aggregates was defined as $\Delta S_{c}=\int_{0}^{b}g\left(x\right)\;dx$ in any increment of time Δt.

Finally, the total mass loss caused by molten and cutting in any increment of time Δt is

(37) $$\Delta M_{i}=\rho_{p}\pi \int_{0}^{b}{\left(\Delta S_{m}+\Delta S_{c}\right)}^{2}\;dx$$

Based on the above analysis, the total time of penetration process was divided into *n* parts with each tiny increment of time Δt. The movement distance and mass loss of projectile were calculated in each tiny increment of time Δt. Then, the ultimate depth of penetration and total mass loss were obtained. More specifically, the profile of the nose was divided into enough grid nodes and the entire section of the projectile was meshed based on those gird nodes, as shown in Figure 6. The two-dimensional alternating direction implicit method was used to calculate the temperature of each node on the projectile mesh. After that, various parameters, such as profile of nose, instantaneous mass, instantaneous velocity, instantaneous deceleration, and the movement distance of projectile, were updated after calculating the mass loss in each time step. Finally, the iterative calculation was established to obtain the results of penetration, such as depth of penetration, mass loss, and shape of the projectile nose. The details of the calculation are shown in Algorithm 2. On the one hand, the temperature of the surface material determines the yield strength of the surface material, and thus affects the incremental mass abrasion caused by cutting of the hard particles. On the other hand, incremental mass abrasion caused by cutting determines the outline of the projectile nose at the next time step. However, the shape of nose is an important factor influencing the resistance of projectile nose based on dynamic cavity theory, which also affects the friction heat and finally affects the temperature of the surface material at the next time step (see Equation (35)). In other words, the incremental mass abrasion caused by thermal melting and that caused by cutting interacted with each other and were calculated at the same time in the penetration model with abrasion, which is consistent with the conclusion that mass abrasion is coupled by thermal melting and cutting.
**Algorithm 2** Calculation of mass loss in any increment of time Δt.**Require:** the motion parameters and thermodynamic parameters of projectile: $\rho_{p}$, $c_{p}$, *Y*, λ, $T_{m}$, $V_{z}$, φ;**Ensure:** the total mass loss caused by molten and cutting in any increment of time Δt;Traversal all valid nodes on the profile of nose and mark the minimum and maximum as $i_{min}$ and $i_{max}$, respectively;2:Given the value of $V_{z}$ and φ;**for**$i=i_{min}$ to $i_{max}$ **do**;4:    Calculate the stress $\sigma_{r}$ on the surface of the projectile nose at the node *i*. Then, calculate the heat flux density of boundary *Q* at node *i*;    Based on the two-dimensional heat conduction and the heat flux density of boundary *Q*, determine the value of area where the temperature exceeds the melting point of projectile material;6:    Calculate the area cutting by aggregates by Equation (36);**end for**8:Calculate the total mass loss in increment of time Δt based on Equation (37);Update the motion parameters of projectile.

## 4. Results and Discussion

### 4.1. Comparison of the Predicted Results with the Experimental Data

Table 2 shows the comparison of DOP and mass loss rate between the proposed model and experimental data. The error between ultimate DOP predicted by the proposed model and experimental data is less than 7.0%, except for the initial impact velocity at 1386.0 m/s. The initial impact velocity of projectile No. 2 is similar to that of projectile No. 3, but the DOP of projectile No. 3 is significantly lower than that of projectile No. 2 and even lower than that of projectile No. 1 which has the lowest initial impact velocity. The possible reason is error in the measurement of DOP or the trajectory was not perfectly straight. Besides, the error between mass loss rate predicted by the proposed model is less than 15.0%. The above discussion shows that the predicted results by the proposed model are in good agreement with the experimental results.

**Figure 7:**
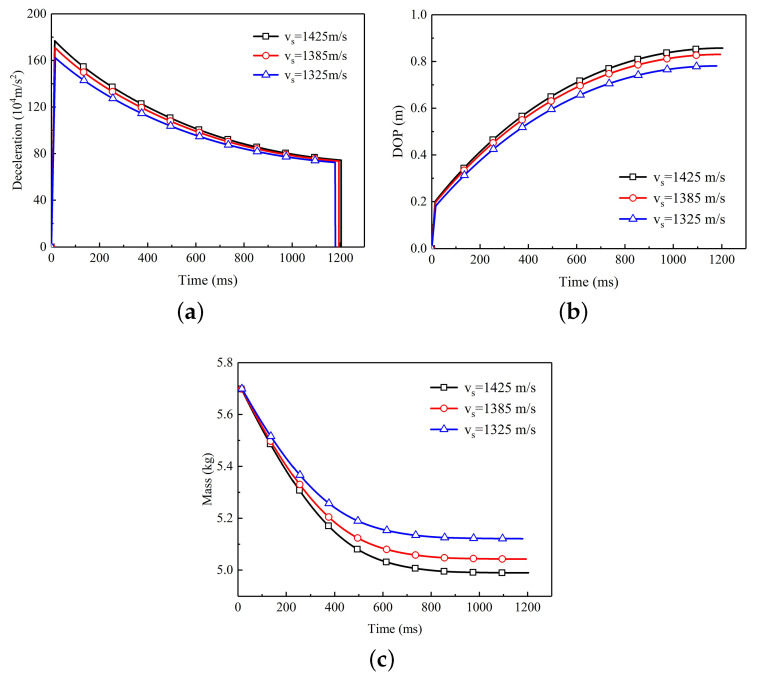
The variation of various parameters of the projectile versus time. (**a**) Relationship between deceleration and time. With the increase of penetration time, the deceleration decreases gradually. (**b**) Relationship between depth of penetration and time. With the increase of penetration time, the depth of penetration increases gradually. (**c**) Relationship between residual mass and time. With the increase of penetration time, the residual mass of projectiles decrease gradually and finally remains constant at the final stage.

Figure 7 shows the variation of various parameters of the projectile versus time during the penetration process. According to the dynamic cavity expansion theory, the deceleration reduction of the projectile is related to velocity. The deceleration of projectile also continues to decrease and finally tends to a fixed value with the increase of time, as shown in Figure 7a. Figure 7b shows that the projectile with higher initial impact velocity has a deeper penetration depth. The penetration depth of the projectile increased rapidly in the initial stage of penetration and it gradually stabilized at a constant value with the increase of penetration time. Similarly, the mass of the projectile decreases rapidly at the initial stage of penetration, and it no longer decreases when the penetration is long enough, as shown in Figure 7c.

**Figure 8:**
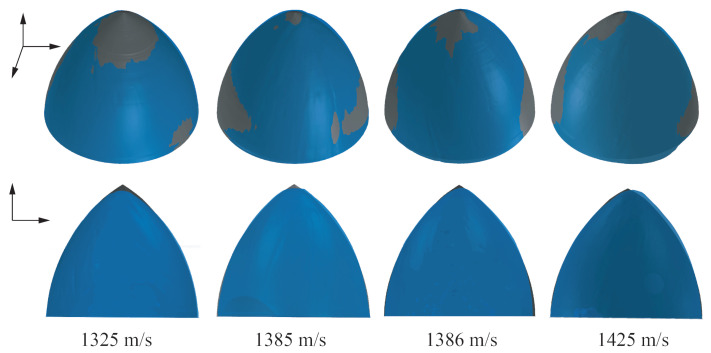
Comparison of the nose shape predicted by the proposed model with the 3D scan photograph of residual projectile. The top four photographs show a comparison between the opaque projectile nose predicted by the proposed model and the 3D scan photograph of the residual projectile. The bottom four photographs show the comparison between the translucent projectile nose predicted by the proposed model and corresponding 2D photograph of the residual projectile.

The recovered projectiles were subjected to three-dimensional (3D) scanning, and the final nose shapes were obtained. Figure 8 shows a comparison between the projectile profile predicted by the proposed method and the photographs of the residual projectile. The top four photographs show a comparison between the opaque projectile nose predicted by the proposed model and the 3D scan photograph of the residual projectile, while the bottom four photographs show the comparison between the translucent projectile nose predicted by the proposed model and corresponding 2D photograph of the residual projectile. The nose shape predicted by the proposed model is in good agreement with the profile of the residual projectile. However, concrete is a complex anisotropic material and aggregates were randomly distributed in the concrete target. Therefore, most of the residual projectiles were not axisymmetric. This is why the area of the two-dimensional sections of the residual projectile nose at a certain angle is less than that predicted by the proposed model; however, the experimental mass loss values are less than those predicted by the proposed model.

**Figure 9:**
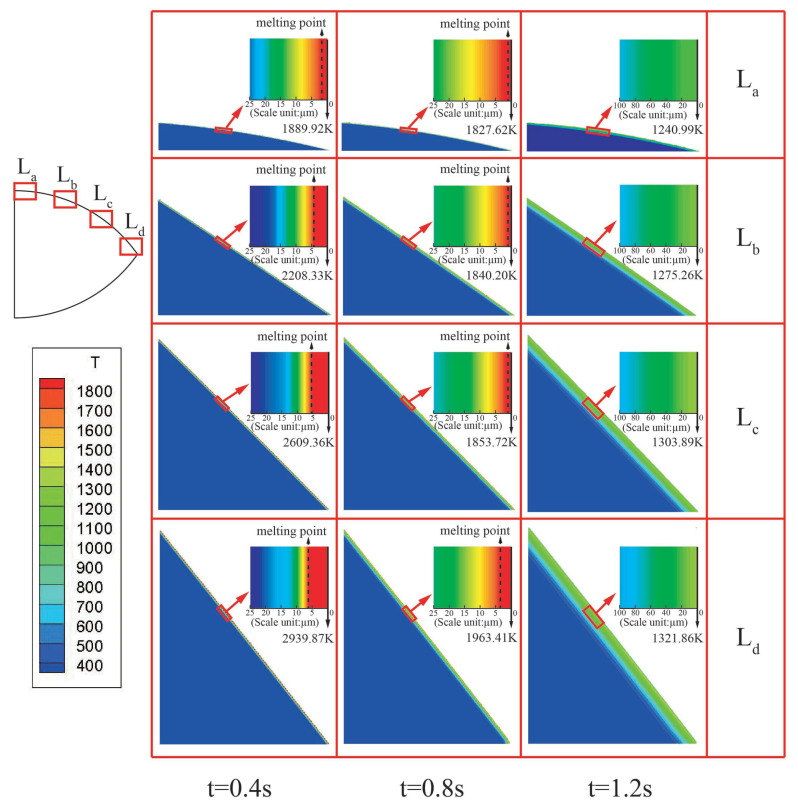
The temperature distribution on projectile surface at different penetrating times. The HAZ thickness at the tip of the nose is obviously larger than that at the tail of the nose.

Furthermore, taking $V_{s}=$ 1385 m/s in Case 2 for analysis, four regions labeled as $L_{a}$, $L_{b}$, $L_{c}$, and $L_{d}$, located from the tip to the end of projectile nose, as shown in top left of Figure 9, were selected to analyze the temperature distribution on projectile surface during the penetration process.

Figure 9 shows that the HAZ thickness at the tip of the nose is larger than that at the tail of the nose. The stress on the nose tip is the largest and the heat generated by friction between the projectile and the target is the greatest at that location. Hence, the thickness of the HAZ at the nose tip is the largest when compared with that of other regions at the same time. Furthermore, with the increase in penetration time, the thickness of the HAZ increases at all the regions of the nose. This is mainly because the friction heat on the projectile surface and the depth of the transferred friction heat increase with the penetration time. However, the maximum temperature on the projectile surface decreases when the penetration time increases. Comparing the temperature distribution of $L_{a}$, $L_{b}$, $L_{c}$, and $L_{d}$ at *t* = 0.4 s, *t* = 0.8 s, and *t* = 1.2 s, the thickness of heat affect zone of different regions on nose increases with the penetration time. At the end of penetration process, i.e., *t* = 1.2 s, the maximum temperatures of the four regions were lower than the melting point. This is because the projectile impact velocity and the friction heat at different regions of the projectile nose drops to a significantly low value at the end of the penetration process. Referring to Figure 7c, the mass loss was approximately zero at *t* = 1.2 s.

### 4.2. Comparison of the Predicted Results with Published Experimental Data and Other Abrasion Models

Forrestal performed a series of penetration tests [13,14]. Those published penetration test data are widely used to verify the validity of the theoretical models, such as Guo’s model [44] and Chen’s model [22]. To further verify the validity of the theoretical model in this section, four groups impact test results in [13] were also collected; the projectiles used in the penetration test normally penetrated into concrete targets with density 2300.0 kg/m^3^. The unconfined compressive strength of concrete target is 51.0 MPa. The initial mass, diameter, and CRH of the projectile are 1.6 kg, 30.5 mm, and 3.0, respectively. Then, the predicted results of the proposed model are compared with other theoretical models and test data in this section.

**Figure 10:**
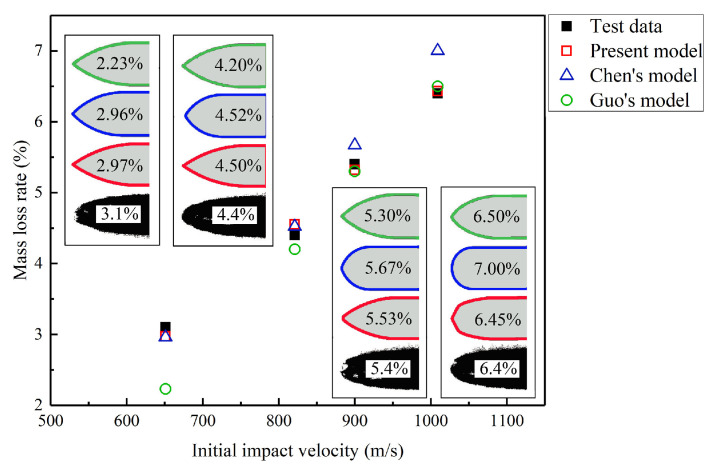
Relationship between impact velocity and mass loss rate. The proposed model agreed well with the experimental data and the residual projectile with different color contour lines represents the predictions by different models.

Generally, the mass abrasion of the projectile is mainly represented by the mass loss rate and nose blunting. Figure 10 shows the comparison of the mass loss rates predicted by different models with published experimental data. The nose profiles predicted by the different models agree well with the experimental data during low-speed penetration. With the initial impact velocity increasing, the mass loss rate predicted by the proposed model is in higher agreement with the experimental data than the other models. Although a large error between the mass loss rate predicted by Chen’s model and most of the experimental data is not observed, Chen’s model contains a fitting parameter that refers to Silling [20] which limits its application. The mass loss rate predicted by Guo’s model is significantly lower than the experimental value at the initial impact velocity of 651 m/s, and the corresponding error is 28.0%. However, the results predicted by Guo’s model are significantly higher than the experimental data at the initial impact velocity of 1201 m/s, and the corresponding error is 26.0%. Therefore, Guo’s model only achieves accurate prediction results within the initial impact velocity range of 800–1000 m/s, and the prediction results at low and high initial impact velocities are significantly different from the experimental data. The main reason for this is that Guo’s model considers a single mass abrasion mechanism. Figure 10 also shows a comparison between the theoretical prediction results by different models and experimental results considering the variation of the projectile nose shape. The profile of the projectile nose predicted by the proposed model, Chen’s model, and Guo’s model were similar at initial impact velocities less than 800 m/s. With increasing the impact velocity, Chen’s model considers that the residual ogive-nose becomes significantly blunter and the corresponding value of CRH also becomes smaller; therefore, the nose shape predicted by Chen’s model for high-speed penetration resulted excessively blunt compared with that of the experimental residual projectile. The residual ogive-nose always varies between the original geometry and a semi-spherical nose; particularly, the length of the residual shanks does not reduce. Conversely, the front of the projectile nose predicted by Guo’s model remains pointed with the impact velocity increasing. Compared with the results of the other two models, the nose shape predicted by the proposed model is more consistent with the experimental results for high-speed penetration. Particularly, the blunting of the nose tip predicted by the proposed model is closer to that of the real of residual projectile.

**Figure 11:**
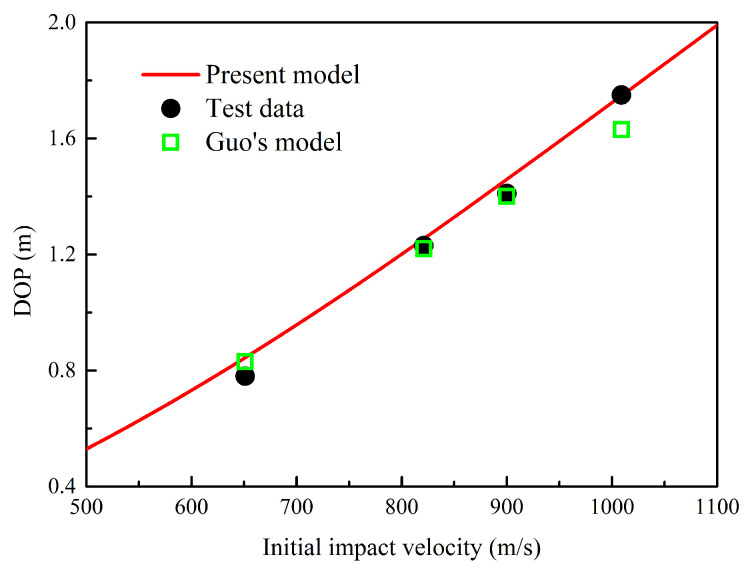
Relationship between impact velocity and DOP. The proposed model agreed well with the experimental data, especially under the condition of high-speed penetration.

Furthermore, the comparison between the DOP predicted by different theoretical models and experimental results is shown in Figure 11. Since Chen did not predict the DOP in reference [22], the DOP predicted by the proposed modell and Guo’s model were compared with experimental data. The results predicted by two models are relatively close, and they both agreed well with experimental data when initial impact velocity is less than 900.0 m/s. However, the DOP predicted by Guo’s model was less than test data during high-speed impact. In summary, the proposed model had better agreement with test data during both low- and high-speed impact tests.

The two main mass abrasion mechanisms, melting and cutting, are coupled in the proposed model. Therefore, the limitations of the previous studies, which considered single abrasion mechanisms, are overcome. Furthermore, the proposed model can simulate the phenomenon of mass abrasion of the projectile body and predict the penetration performance of projectiles at high initial impact velocities more accurately.

## 5. Conclusions

Mass abrasion and nose blunting may result in the structural failure of a projectile and significantly affect the penetration performance of a projectile. In this study, experimental investigations on projectiles under impact loading were conducted to further explore the mechanism of structural failure caused by mass abrasion and nose blunting. Based on the microscopic observations of residual projectiles, melting and cutting are two main failure mechanisms during the high-speed impact and a theoretical model with coupled mechanisms was proposed to predict failure characteristics and penetration performance of projectile more reasonably.

(1) Experiments on projectiles under high-speed impact loading were conducted and the experimental data, such as DOP and mass loss, were obtained. Furthermore, the microscopic observations of the residual projectiles indicated that projectile surface thermal melting and cutting by the aggregates are the two main failure mechanisms simultaneously during high-speed impact into concrete slabs.

(2) A nonlinear failure criterion, modified cap model, and a three-stage HJC equation of state were introduced in dynamic cavity expansion theory owing to better applicability to high-speed penetration. Then, a theoretical model with coupled mechanisms were proposed to predict the mass abrasion and nose blunting during high-speed impact into concrete slabs.

(3) Compared with previous theoretical models, which generally focus on a single abrasion mechanism, the coupled melting–cutting theoretical model proposed herein was more consistent with the experimental data at high initial impact velocities. The parameters representing penetration performance and failure characteristics of a projectile during high-speed impact into concrete slabs, such as DOP, mass loss, and the variation of the projectile nose, could be predicted by the proposed model and agreed well with the experimental data.

## Figures and Tables

**Table 1:** The parameters of concrete used in calculation.

$f_{c}/\left(\mathrm{MPa}\right)$	$f_{t}/\left(\mathrm{MPa}\right)$	$p_{m}/\left(\mathrm{MPa}\right)$	$p_{l}/\left(\mathrm{MPa}\right)$	$\kappa_{e}$	$\kappa_{p}$	***R***	$\rho_{0}/\left(\mathrm{kg}/m^{3}\right)$
50.0	4.0	476.0	800.0	0.0013	0.11	0.91	2300.0
$K_{c}/\left(\mathrm{GPa}\right)$	$K_{l}/\left(\mathrm{GPa}\right)$	$A^{\prime }$	$k_{1}$	$k_{2}$	$B^{\prime }$	ν	E/(GPa)
7.18	52.8	1.8076	14.4863	0.9914	4.0962	0.22	209

**Table 2:** Comparison of predicted result with the experimental data.

Test No.	Initial Impact Velocity (m/s)	DOP (m)	Test Data (m)	Error	Mass Loss Rate (%)	Test Data (%)	Error
No. 1	1325	0.79	0.74	6.7%	9.6	9.05	6.1%
No. 2	1385	0.83	0.82	1.2%	10.4	9.06	14.8%
No. 3	1386	0.83	0.70	18.6%	10.5	9.77	7.5%
No. 4	1425	0.86	0.86	0.1%	11.4	10.42	9.4%

## Abbreviations

The following abbreviations are used in this manuscript:

SEM	Scanning electron microscopy
DOP	Depth of penetration
HAZ	Heat affected zone
CRH	Caliber-radius-head

## References

[1] Xiao X.K., Mu Z.C., Pan H., Lou Y.S. (2018). Effect of the Lode parameter in predicting shear cracking of 2024-T351 aluminum alloy Taylor rods. Int. J. Impact Eng..

[2] Hamed S., Keith D., Rooholamin D., Abolgazl D. (2019). Scaled models for failure under impact loading. Int. J. Impact Eng..

[3] Ning J., Chen L. (2004). Fuzzy interface treatment in Eulerian method. Sci. China Ser. E Technol. Sci..

[4] Liu B., Guedes C. (2015). Plastic response and failure of rectangular cross-section tubes subjected to transverse quasi-static and low-velocity impact loads. Int. J. Impact Eng..

[5] Li J., Hao L., Li J. (2019). Theoretical modeling and numerical simulations of plasmas generated by shock waves. Sci. China Technol. Sci..

[6] Rosenberg Z., Vayig Y. (2020). The scaling issue in the penetration of concrete targets by rigid projectiles—Revisited. Int. J. Impact Eng..

[7] Ning J., Song W., Yang G. (2006). Failure analysis of plastic spherical shells impacted by a projectile. Int. J. Impact Eng..

[8] Chen X.W., Li Q.M. (2004). Transition from nondeformable projectile penetration to semihydrodynamic penetration. J. Eng. Mech-ASCE.

[9] Li Q.M., Chen X.W. (2003). Dimensionless formulae for penetration depth of concrete target impacted by a non-deformable projectile. Int. J. Impact Eng..

[10] Forrestal M.J., Altman B.S., Cargile J.D., Hanchak S.J. (1994). An empirical equation for penetration depth of ogive-nose projectiles into concrete targets. Int. J. Impact Eng..

[11] Forrestal M.J., Tzou D.Y. (1997). A spherical cavity-expansion penetration model for concrete targets. Int. J. Solid Struct..

[12] Xu X., Ma T., Liu H., Ning J. (2019). A three-dimensional coupled Euler-PIC method for penetration problems. Int. J. Numer. Methods Eng..

[13] Forrestal M.J., Frew D.J., Hanchak S.J., Brar N.S. (1996). Penetration of grout and concrete targets with ogive-nose steel projectiles. Int. J. Impact Eng..

[14] Frew D.J., Hanchak S.J., Green M.L., Forrestal M.J. (1998). Penetration of concrete targets with ogive-nose steel rods. Int. J. Impact Eng..

[15] Zhang Y.D., Lu Z.C., Wen H.M. (2019). On the penetration of semi-infinite concrete targets by ogival-nosed projectiles at different velocities. Int. J. Impact Eng..

[16] Feng J., Song M., Sun W. (2018). Thick plain concrete targets subjected to high speed penetration of 30CrMnSiNi2A steel projectiles: Tests and analyses. Int. J. Impact Eng..

[17] Yang J.C., Zuo X.J., He X. (2012). Experimental Study of projectile mass loss in high velocity penetration of concrete target. J. Exp. Mech..

[18] Liu C., Zhang X.F., Chen H.H. (2020). Experimental and theoretical study on steel long-rod projectile penetration into concrete targets with elevated impact velocities. Int. J. Impact Eng..

[19] Ning J., Ren H., Guo T., Li P. (2013). Dynamic response of alumina ceramics impacted by long tungsten projectile. Int. J. Impact Eng..

[20] Silling S.A., Forrestal M.J. (2007). Mass loss from abrasion on ogive-nose steel projectiles that penetrate concrete targets. Int. J. Impact Eng..

[21] Wen H.M., Yang Y., He T. (2010). Effects of abrasion on the penetration of ogival-nosed projectiles into concrete targets. Lat. Am. J. Solids Struct..

[22] Chen X.W., He L.L., Yang S.Q. (2010). Modeling on mass abrasion of kinetic energy penetrator. Eur. J. Mech. A Solids.

[23] Jones S.E., Foster J.C., Toness O.A., DeAngelis R.J., Rule W.K. An estimate for mass loss from high velocity steel penetrators. Proceedings of the ASME PVP-435 Conference on Thermal–Hydraulic Problems, Sloshing Phenomena, and Extreme Loads on Structures.

[24] Davis R.N., Neely A.M., Jones S.E. (2004). Mass loss and blunting during high-speed penetration. Proc. Inst. Mech. Eng. Part C J. Mech. Eng. Sci..

[25] He L.L., Chen X.W., He X. (2010). Parametric study on mass loss of penetrators. Acta Mech. Sin..

[26] Ouyang H., Chen X. (2018). Modeling on mass loss and nose blunting of high-speed penetrator into concrete target. Int. J. Prot. Struct..

[27] Guo L., He Y., Zhang N.S., Pang C.X., Hao Z. (2014). On the mass loss of a projectile based on the Archard theory. Explos. Shock Waves.

[28] Ning J., Ma T., Fei G. (2014). Multi-material Eulerian method and parallel computation for 3D explosion and impact problems. Int. J. Comput. Methods.

[29] Luk V.K., Forrestal M.J. (1987). Penetration into semi-infinite reinforced-concrete targets with spherical and ogival nose projectiles. Int. J. Impact Eng..

[30] Meng C.M., Tan Q.H., Jiang Z.G., Song D.Y., Liu F. (2018). Approximate solutions of finite dynamic spherical cavity-expansion models for penetration into elastically confined concrete targets. Int. J. Impact Eng..

[31] Deng Y.J., Song W.J., Chen X.W. (2019). Spherical cavity-expansion model for penetration of reinforced-concrete targets. Acta Mech. Sin..

[32] Ottosen N.S. (1979). A failure criterion for concrete. J. Eng. Mech. Div. ASCE.

[33] Holmquist T.J., Johnson G.R., Cook W.H. A computational constitutive model for concrete subjected to large strains, high strain rate, and high pressures. Proceedings of the 14th International Symposium on Ballistics.

[34] Ren G.M., Wu H., Fang Q., Kong X.Z. (2017). Parameters of Holmquist-Johnson-Cook model for high-strength concrete-like materials under projectile impact. Int. J. Prot. Struct..

[35] Roscoe K.H., Schofied M.A. (1958). On the yielding of soils. Geotechnique.

[36] Sandler I.S. (2005). Review of the development of Cap Models for geomaterials. Shock Vib..

[37] Feng J., Li W., Wang X., Song M., Ren H., Li W. (2015). Dynamic spherical cavity expansion analysis of rate-dependent concrete material with scale effect. Int. J. Impact Eng..

[38] Liu Z.L., Sun W.W., Wang X.M., Feng J. (2015). Spherical cavity-expansion model for concrete targets based on cap model and penetration resistance analysis. Acta Armamentarii.

[39] Hanchak S.J., Forrestal M.J., Young E.R., Ehrgott J.Q. (1992). Perforation of concrete slabs with 48 MPa (7 ksi) and 140 MPa (20 ksi) unconfined compressive strengths. Int. J. Impact Eng..

[40] Gebbeken N., Greulich S., Pietzsch A. (2006). Hugoniot properties for concrete determined by full-scale detonation experiments and flyer-plate-impact tests. Int. J. Impact Eng..

[41] Satapathy S. (2001). Dynamic spherical cavity expansion in brittle ceramics. Int. J. Solids Struct..

[42] He L., Chen X. (2011). Analyses of the penetration process considering mass loss. Eur. J. Mech. A Solids.

[43] Rabinowicz E., Dunn L., Russell P. (1961). A study of abrasive wear under three-body conditions. Wear.

[44] Guo L., He Y., Zhang X., He Y., Deng J., Guan Z. (2017). Thermal-mechanical analysis on the mass loss of high-speed projectiles penetrating concrete targets. Eur. J. Mech. A Solids.

